# Supplementary figures and images for: Tet1 regulates epigenetic remodeling of the pericentromeric heterochromatin and chromocenter organization in DNA hypomethylated cells
Source: PLoS Genet. 2021 Jun 24;17(6):e1009646. doi: 10.1371/journal.pgen.1009646 (PMC8263065; doi:10.1371/journal.pgen.1009646)

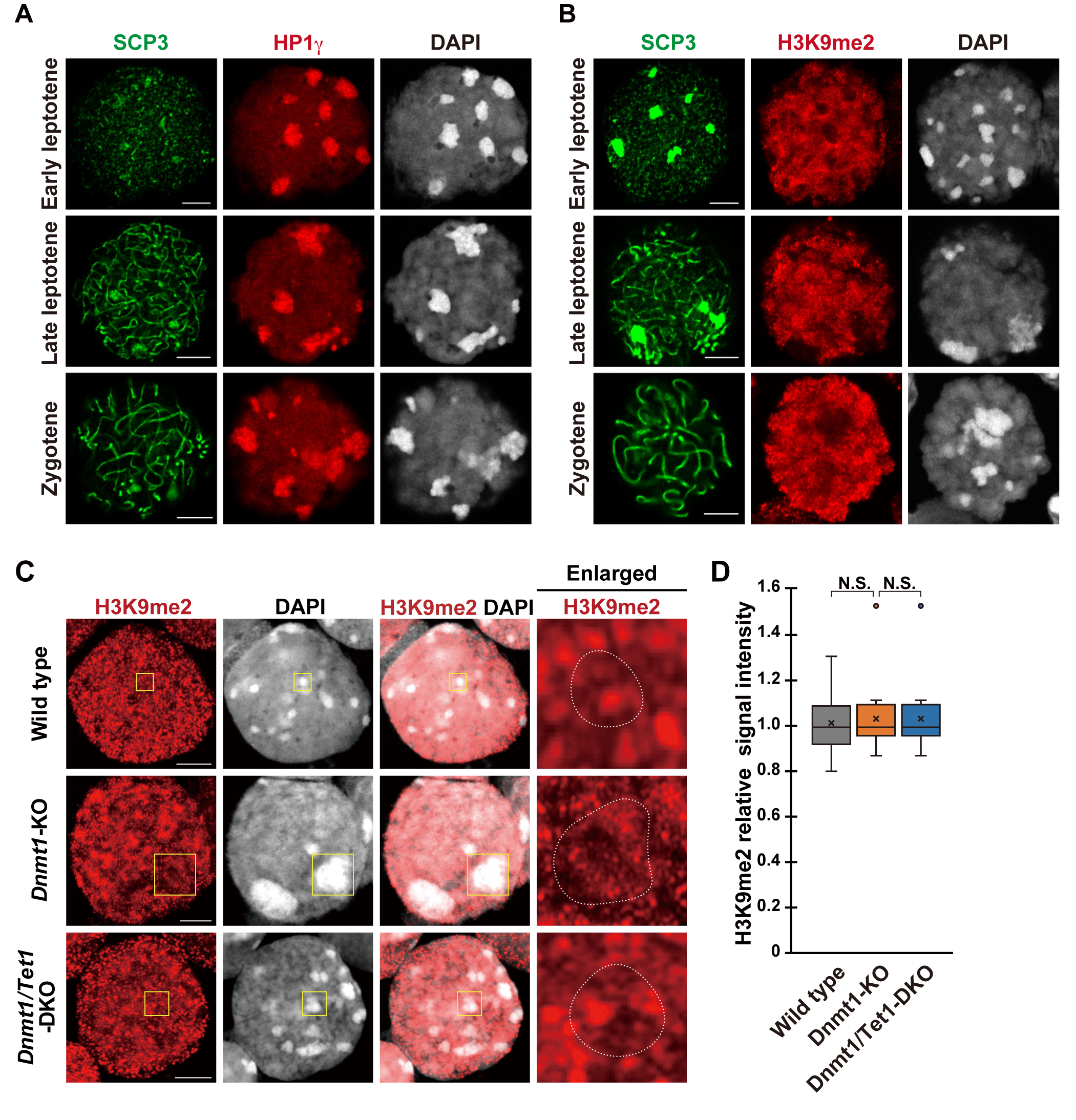

Supplement: S1 Fig — (A), (B): Representative immunostaining images of surface-spread nuclei at the first meiotic prophase of female. Germ cells were harvested from E15.5-E16.5 embryos. (C): Representative immunostaining images of the wild-type, Dnmt1-KO, and Dnmt1/Tet1-DKO ESCs. The yellow squares indicate the enlarged areas shown on the right panels. The white dashed circles indicate the chromocenter. (D): Quantification of H3K9me2 enrichment in the chromocenter, related to (C). The signal intensity at the chromocenter was normalized with whole nuclear area. Wild-type, n = 16; Dnmt1-KO, n = 16; Dnmt1/Tet1-DKO, n = 16. Scale bar, 5 μm. (TIF) [file pgen.1009646.s001.tif]

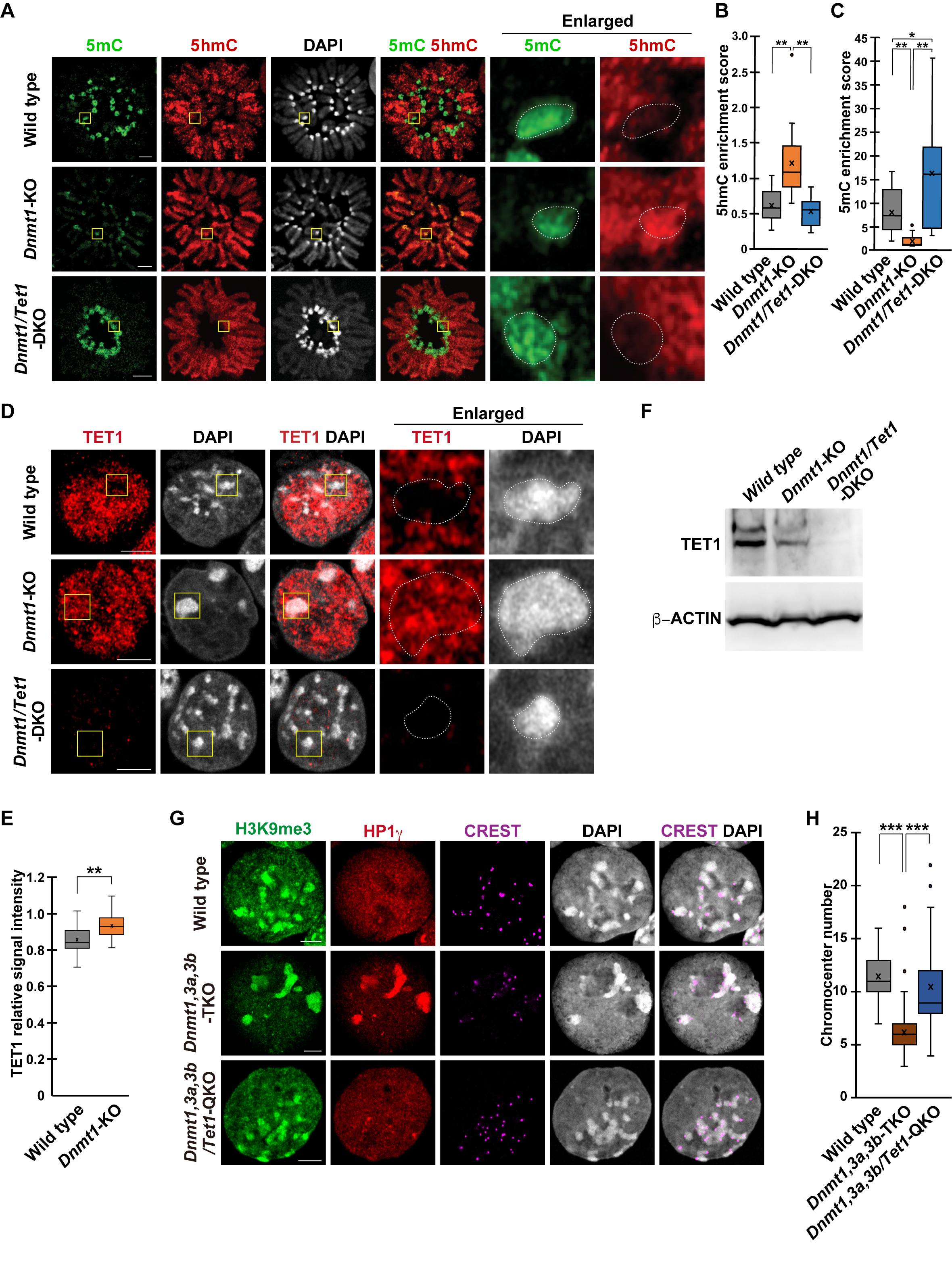

Supplement: S2 Fig — (A): Representative immunostaining images of surface-spread nuclei of the wild-type, Dnmt1-KO and Dnmt1/Tet1-DKO ESCs. The yellow squares indicate the enlarged areas shown on the right. The white dashed circles indicate the PCH. (B), (C): Quantification of immunostaining of 5hmC (B) and 5mC (C), related to (A). The enrichment score is calculated by the relative signal intensity of PCH to the entire chromosome. An average of 3 chromosomes was calculated for each cell. n = 20 per cell line. (D): Representative immunostaining images of the wild-type, Dnmt1-KO and Dnmt1/Tet1-DKO ESCs. The yellow squares indicate the enlarged areas shown on the right. White dashed circles indicate the chromocenter. ESCs were plated in a culture slide and cultured for 1 d before immunostaining. (E): Quantification of TET1 enrichment in the chromocenter, related to (D). The signal intensity at the chromocenter was normalized with whole nuclear area. Wild-type, n = 21; Dnmt1-KO, n = 22. (F): Western blotting of whole cell extract from the wild-type, Dnmt1-KO, and Dnmt1/Tet1-DKO ESCs. (G): Representative immunostaining images of surface-spread nuclei of the wild-type, Dnmt1/3a/3b-TKO, and Dnmt1/3a/3b/Tet1-QKO ESCs. (H): Boxplot showing the number of distinct chromocenters in the nuclei of ESCs, related to (G). Wild-type, n = 55; Dnmt1/3a/3b-TKO, n = 99; Dnmt1/3a/3b/Tet1-QKO, n = 55. P values were calculated using the Mann-Whitney U-test. ***P < 0.001; **P < 0.01; *P < 0.05. Scale bar, 5μm. (TIF) [file pgen.1009646.s002.tif]

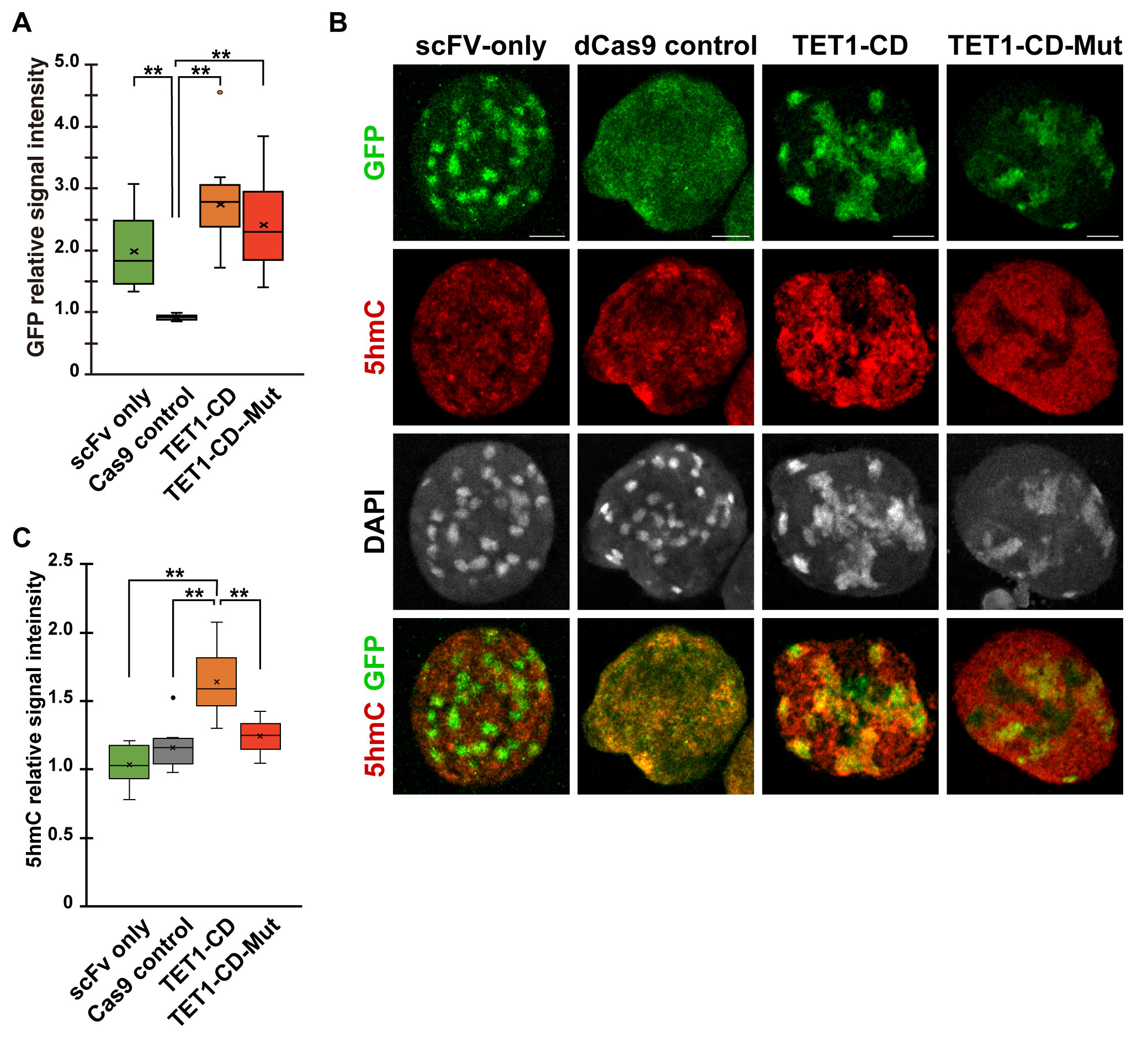

Supplement: S3 Fig — (A): Quantification of GFP enrichment in the chromocenter, related to Fig 2(C). The signal intensity at the chromocenter was normalized with whole nuclear area. scFv-only, n = 10; dCas9 control, n = 12; TET1-CD, n = 15; TET1-CD-Mut, n = 16. (B): Representative 5hmC immunostaining images of the surface-spread nuclei of the ESCs expressing individual cassette. (C): Quantification of immunostaining of 5hmC, related to (B). scFv-only, n = 14 dCas9 control, n = 11; TET1-CD, n = 14; TET1-CD-Mut, n = 11. P values were calculated using the Mann-Whitney U-test. **P < 0.01. Scale bar, 5 μm. (TIF) [file pgen.1009646.s003.tif]

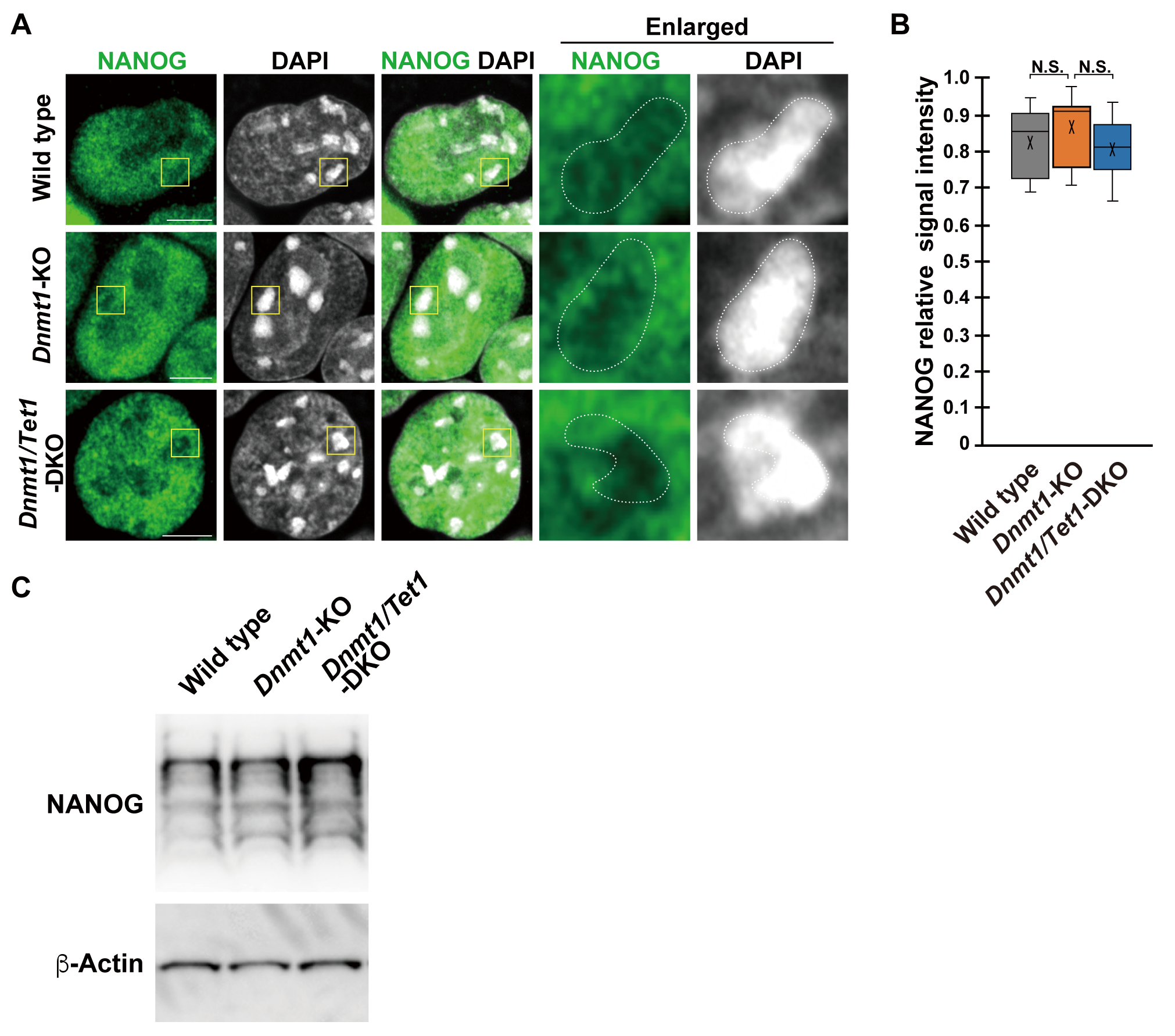

Supplement: S4 Fig — (A): Representative immunostaining images of the wild-type, Dnmt1-KO and Dnmt1/Tet1-DKO ESCs. The yellow squares indicate the enlarged areas shown on the right. White dashed circles indicate the chromocenter. (B): Quantification of NANOG enrichment in the chromocenter, related to (A). The signal intensity at the chromocenter was normalized with whole nuclear area. Wild-type, n = 14; Dnmt1-KO, n = 13; Dnmt1/Tet1-DKO, n = 15. (C): Representative image of Western blotting analysis of whole cell extracts from the wild-type, Dnmt1-KO, and Dnmt1/Tet1-DKO ESCs. (TIF) [file pgen.1009646.s004.tif]

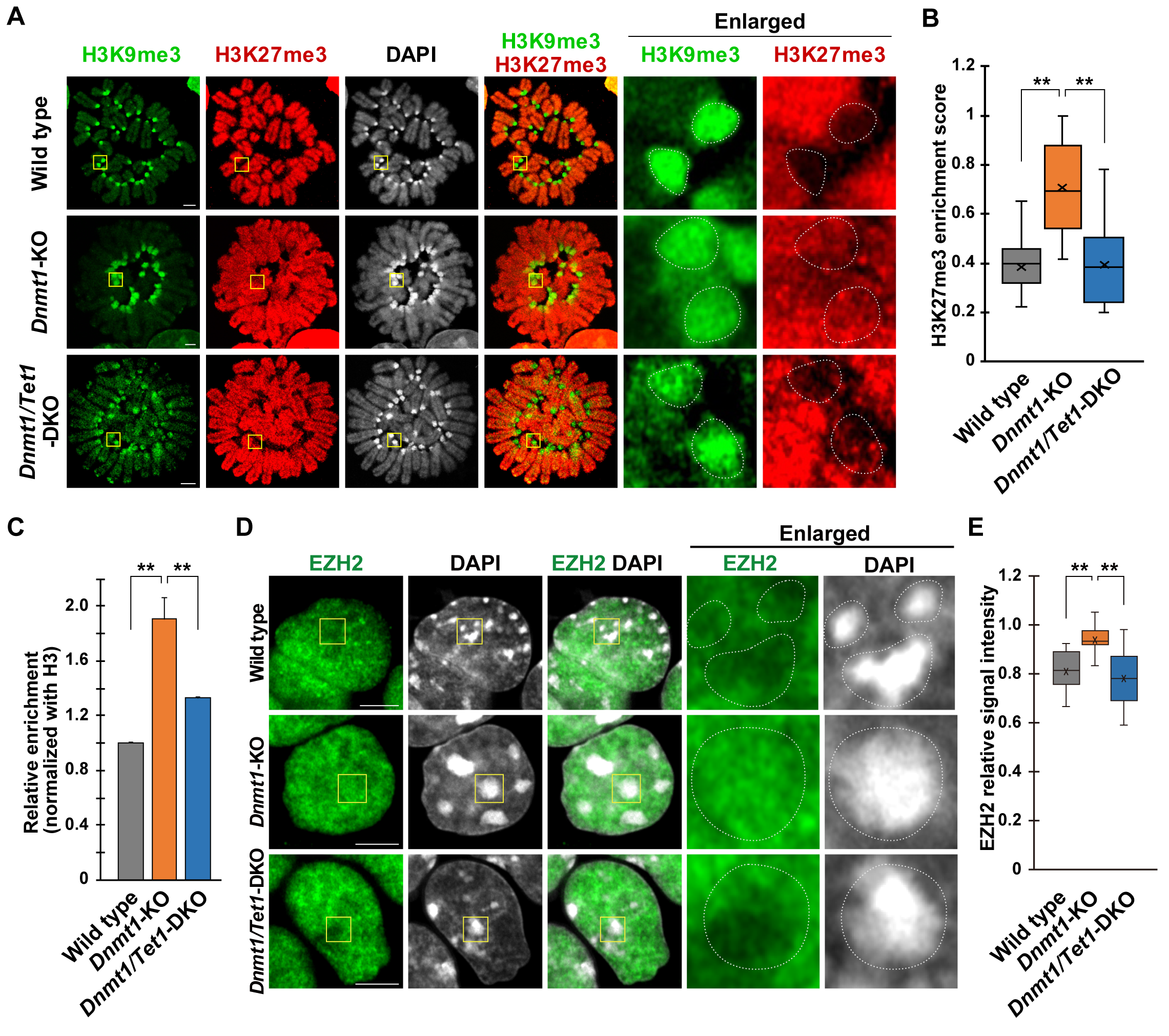

Supplement: S5 Fig — (A): Representative immunostaining images of surface-spread nuclei of the wild-type, Dnmt1-KO and Dnmt1/Tet1-DKO ESCs. The yellow squares indicate the enlarged areas shown on the right. The white dashed circles indicate the PCH. (B): Quantification of immunostaining of H3K27me3, related to (A). n = 20 per cell line. (C): ChIP-qPCR for H3K27me3 at the major satellite repeats in the wild-type, Dnmt1-KO and Dnmt1/Tet1-DKO ESCs. Each bar represents relative enrichment after normalization against H3 ChIP. n = 3 per cell line. (D): Representative immunostaining images of the wild-type, Dnmt1-KO and Dnmt1/Tet1-DKO ESCs. The yellow squares indicate the enlarged areas shown on the right. White dashed circles indicate the chromocenter. ESCs were plated in a culture slide and cultured for 1 d before immunostaining. (E): Quantification of EZH2 enrichment in the chromocenter, related to (D). The signal intensity at the chromocenter was normalized with whole nuclear area. Wild-type, n = 15; Dnmt1-KO, n = 16; Dnmt1/Tet1-DKO, n = 16. P values were calculated using the Mann-Whitney U-test. **P < 0.01. Scale bar, 5 μm. (TIF) [file pgen.1009646.s005.tif]

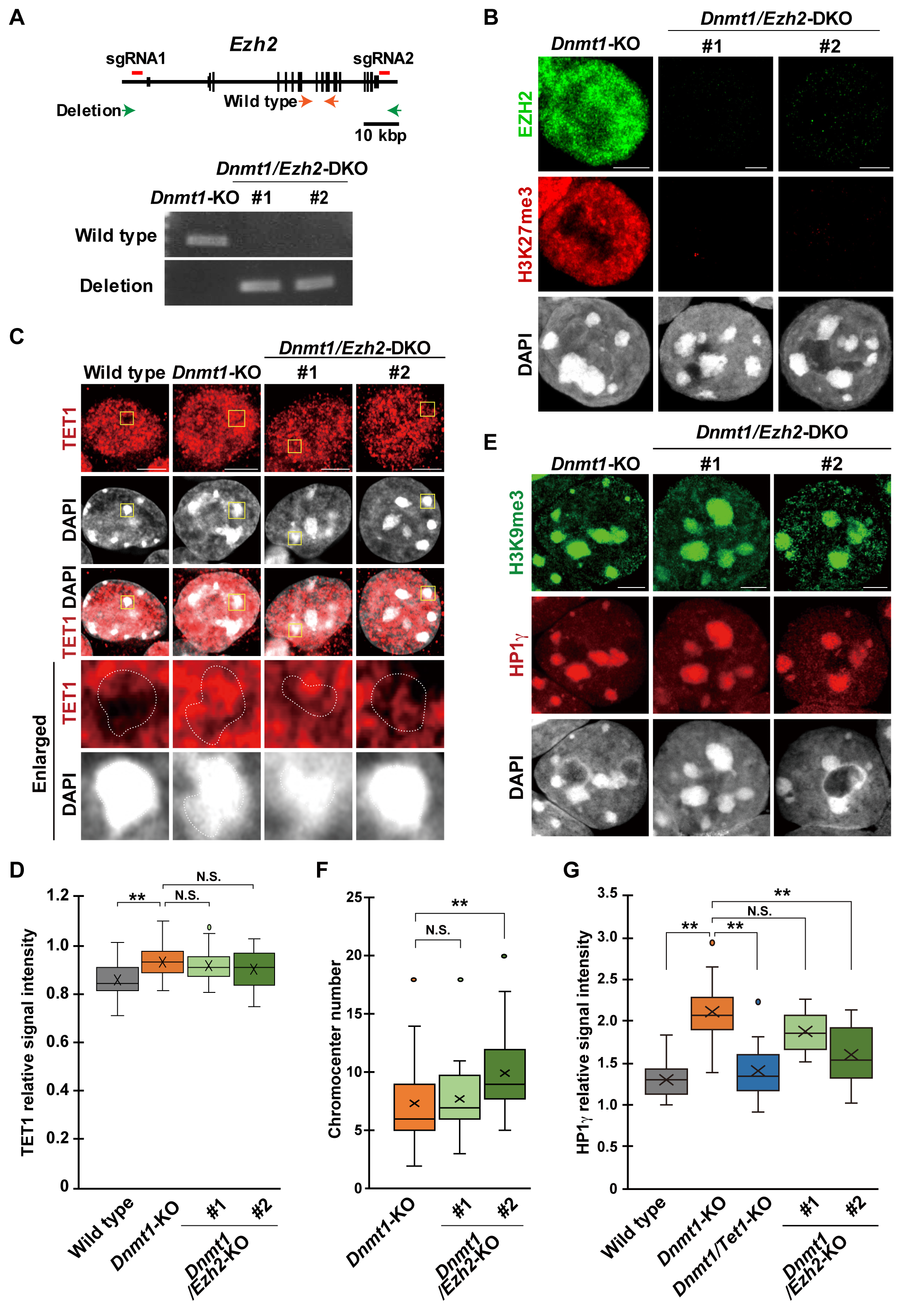

Supplement: S6 Fig — (A): Schematic illustration showing the designs of sgRNA and genotyping PCR for the depletion of Ezh2 (top). Results of genotyping PCR confirming the successful deletion of whole Ezh2 gene locus. (B): Representative immunostaining images of surface-spread nuclei of the Dnmt1-KO and Dnmt1/Ezh2-DKO ESCs. (C): Representative immunostaining images of the wild-type, Dnmt1-KO, and Dnmt1/Ezh2-DKO ESCs. The yellow squares indicate the enlarged areas shown on the right. White dashed circles indicate the chromocenter. ESCs were plated in a culture slide and cultured for 1 d before immunostaining. (D): Quantification of TET1 enrichment in the chromocenter, related to (C). The signal intensity at the chromocenter was normalized with whole nuclear area. Wild-type, n = 21; Dnmt1-KO, n = 22; Dnmt1/Ezh2-DKO #1, n = 19; Dnmt1/Ezh2-DKO #2, n = 23. (E): Representative immunostaining images of surface-spread nuclei of the Dnmt1-KO and Dnmt1/Ezh2-DKO ESCs. (F): Boxplot showing the number of distinct chromocenters in the nuclei of ESCs. Dnmt1-KO, n = 55; Dnmt1/Ezh2-DKO #1, n = 32; DKO #2, n = 37. (G): Quantification of HP1γ enrichment in the chromocenter, related to (E) and Fig 1(A). The signal intensity at the chromocenter was normalized with whole nuclear area. Wild-type, n = 29; Dnmt1-KO, n = 14; Dnmt1/Tet1-DKO, n = 18; Dnmt1/Ezh2-DKO #1, n = 16; Dnmt1/Ezh2-DKO #2, n = 16. P values were calculated using the Mann-Whitney U-test. N.S, no significance, P > 0.05; **P < 0.01. Scale bar, 5 μm. (TIF) [file pgen.1009646.s006.tif]

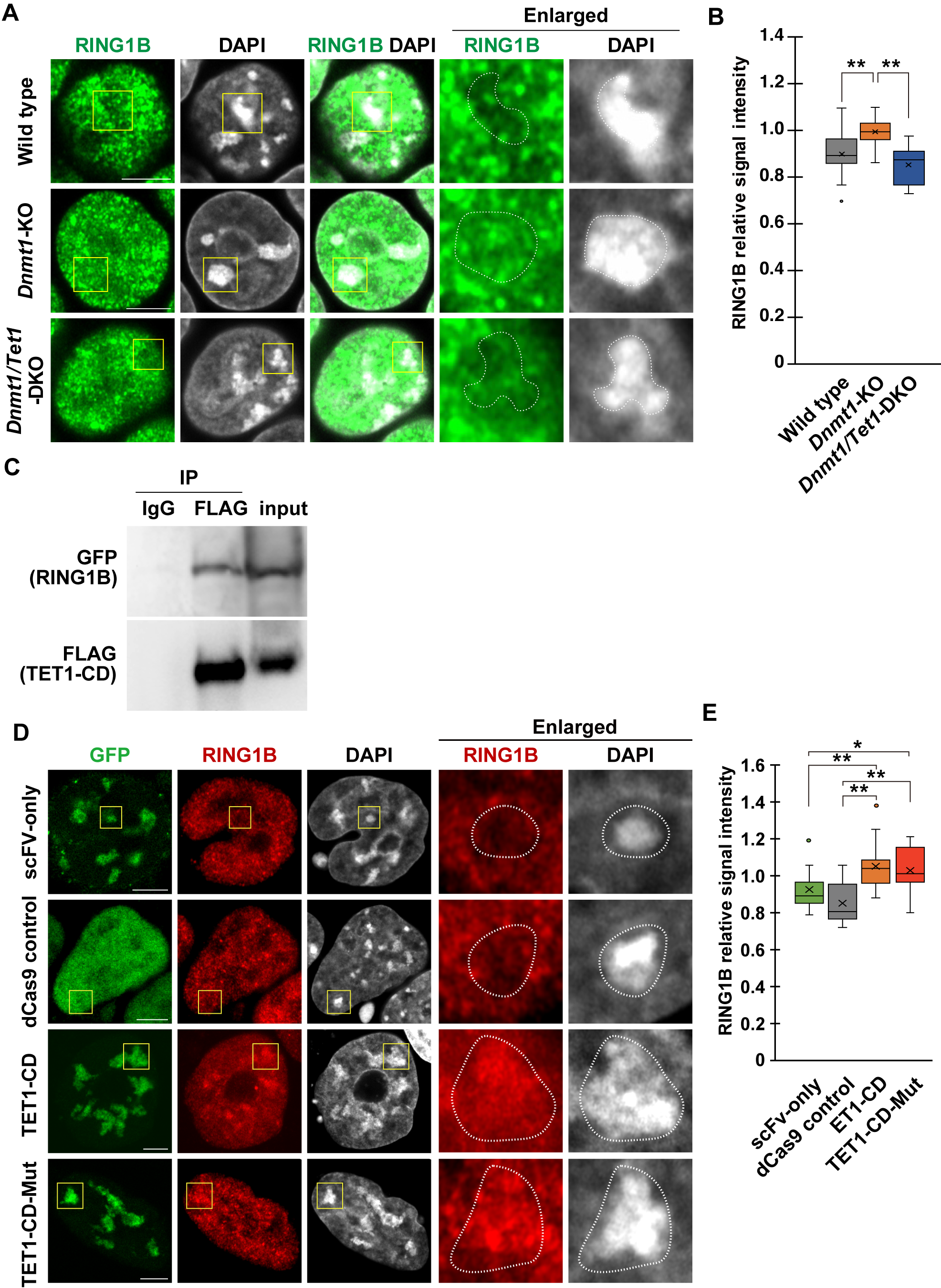

Supplement: S7 Fig — (A): Representative immunostaining images of the wild-type, Dnmt1-KO, Dnmt1/Tet1-DKO, and Dnmt1/Ezh2-DKO ESCs. Yellow squares indicate the enlarged areas shown on the right. White dashed circles indicate the chromocenter. The ESCs were plated in a culture slide and cultured for 1 d before immunostaining. Scale bar, 5 μm. (B): Quantification of RING1B enrichment in the chromocenter, related to (A). The signal intensity at the chromocenter was normalized with whole nuclear area. Wild-type, n = 19; Dnmt1-KO, n = 21; Dnmt1/Tet1-DKO, n = 22. (C): Co-immunoprecipitation analysis of TET1 and RING1B. Cell lysate were immunoprecipitated with anti-IgG or anti-DDDDK-tag (FLAG) antibodies. Western blotting was performed using the antibodies indicated. (D): Representative immunostaining images of surface-spread nuclei of the ESCs expressing scFv-only, dCas9 control, TET1-CD, and TET1-CD-Mut cassette. Yellow squares indicate the enlarged areas shown on the right. White dashed circles indicate the chromocenter. (E): Quantification of RING1B enrichment in the chromocenter, related to (D). The signal intensity at the chromocenter was normalized with whole nuclear area. scFv-only, n = 10; dCas9 control, n = 12; TET1-CD, n = 15; TET1-CD-Mut, n = 16. P values were calculated using the Mann-Whitney U-test. **P < 0.01. *P < 0.05. Scale bar, 5 μm. (TIF) [file pgen.1009646.s007.tif]

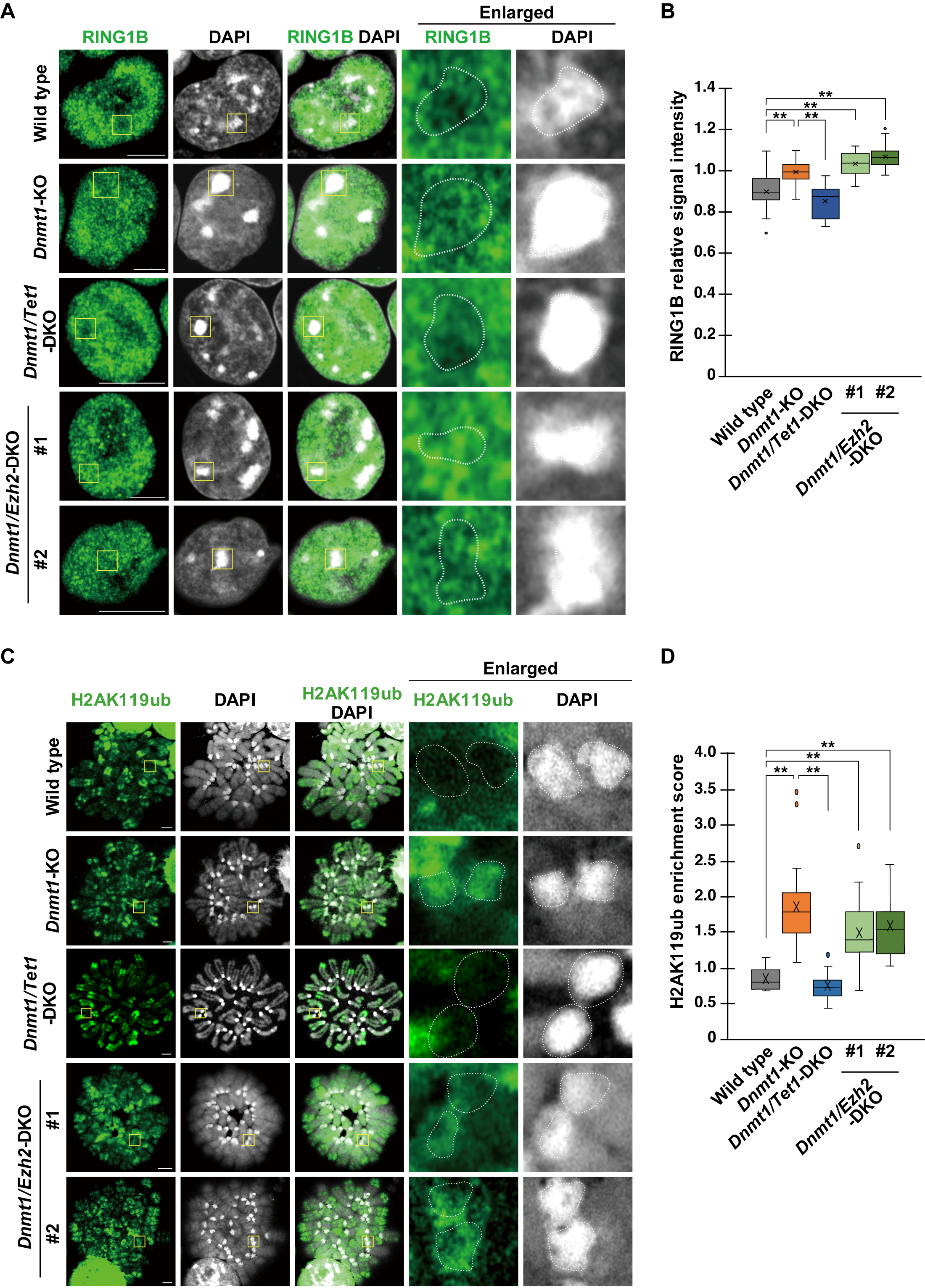

Supplement: S8 Fig — (A): Representative immunostaining images of the wild-type, Dnmt1-KO, Dnmt1/Tet1-DKO, and Dnmt1/Ezh2-DKO ESCs. Yellow squares indicate the enlarged areas shown on the right. White dashed circles indicate the chromocenter. The ESCs were plated in a culture slide and cultured for 1 d before immunostaining. Scale bar, 5 μm. (B): Quantification of RING1B enrichment in the chromocenter, related to (A). The signal intensity at the chromocenter was normalized with whole nuclear area. Wild-type, n = 19; Dnmt1-KO, n = 21; Dnmt1/Tet1-DKO, n = 22; Dnmt1/Ezh2-DKO #1, n = 22; Dnmt1/Ezh2-DKO #2, n = 25. (C): Representative immunostaining images of surface-spread nuclei of the wild-type, Dnmt1-KO and Dnmt1/Ezh2-DKO ESCs. The yellow squares indicate the enlarged areas shown on the right. The white dashed circles indicate the PCH. (D): Quantification of immunostaining of H2AK119ub, related to (C). n = 20 per cell line. P values were calculated using the Mann-Whitney U-test. **P < 0.01. Scale bar, 5 μm. (TIF) [file pgen.1009646.s008.tif]

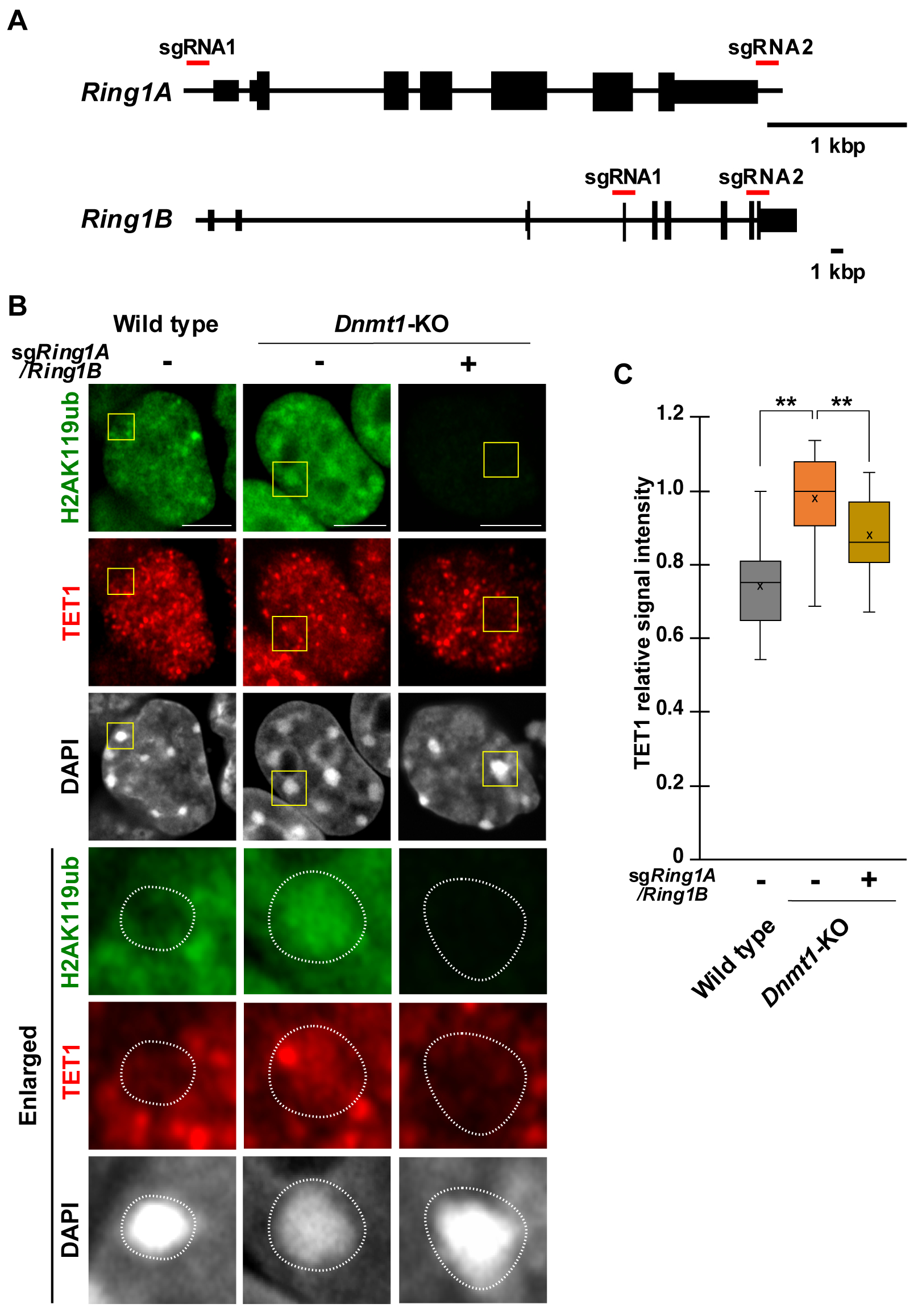

Supplement: S9 Fig — (A): Schematic illustration showing the designs of sgRNA for the depletion of Ring1A (top) and Ring1B (bottom). (B): Representative immunostaining images of surface-spread nuclei of the wild-type, Dnmt1-KO and Dnmt1/Ring1A/1B-TKO ESCs. Cells were analyzed 4 d after the transfection of the CRISPR cassette. (C): Quantification of TET1 enrichment in the chromocenter, related to (B). The signal intensity at the chromocenter was normalized with whole nuclear area. Wild-type, n = 20; Dnmt1-KO, n = 22; Dnmt1/Ring1A/1B-TKO, n = 22. P values were calculated using the Mann-Whitney U-test. **P < 0.01. Scale bar, 5 μm. (TIF) [file pgen.1009646.s009.tif]

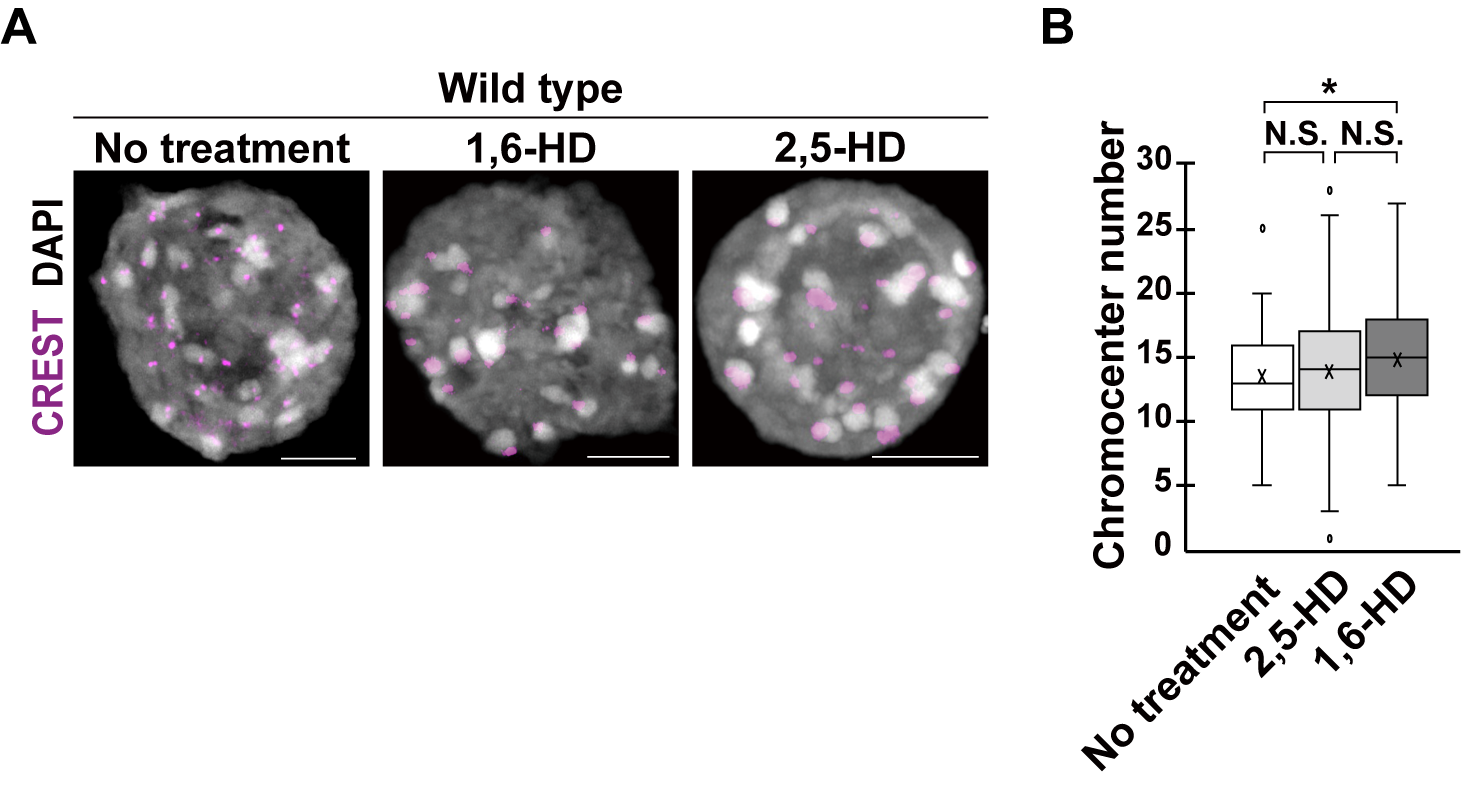

Supplement: S10 Fig — (A): Representative immunostaining images of surface-spread nuclei of the wild-type ESCs with or without HD treatment. About 10% of 2,5- or 1,6-HD were treated for 1 min. (B): Boxplot showing the number of distinct chromocenters in the nuclei of wild-type ESCs treated with 2,5- or 1,6-HD, related to (A). No treatment, n = 111; 2,5-HD treatment, n = 179; 1,6-HD treatment, n = 105. P values were calculated using the Mann-Whitney U-test. N.S., no significant, P > 0.05. *P < 0.05. (TIF) [file pgen.1009646.s010.tif]
