# Supplementary material for: Tet1 regulates epigenetic remodeling of the pericentromeric heterochromatin and chromocenter organization in DNA hypomethylated cells
Source: PLoS Genet. 2021 Jun 24;17(6):e1009646. doi: 10.1371/journal.pgen.1009646 (PMC8263065; doi:10.1371/journal.pgen.1009646)
Supplement: S1 Table — (DOCX) [file pgen.1009646.s012.docx]

| **S1 Table. sgRNA sequences** | | |
| --- | --- | --- |
| Target | Sequence (5' to 3') | |
| *Tet1* | GGCTGCTGTCAGGGAGCTCA | |
| Major satellite | GAAATGTCCACTGTAGGACG | |
| *Ezh2* #1 | AGTATGGCACGCCGGAGAAG | |
| *Ezh2* #2 | TCCCCAGGGTACAAGTTGGC | |
| *Ring1a* #1 | TGCAGCTGCGGCCGGCTTTC | |
| *Ring1a* #2 | TCACCTTCCTATTTACAACC | |
| *Ring1b* #1 | TCAACCATTAAGCAAAACAT | |
| *Ring1b* #2 | AAAAGCTCATTTGTGCTCCT | |
