# Supplementary material for: Tet1 regulates epigenetic remodeling of the pericentromeric heterochromatin and chromocenter organization in DNA hypomethylated cells
Source: PLoS Genet. 2021 Jun 24;17(6):e1009646. doi: 10.1371/journal.pgen.1009646 (PMC8263065; doi:10.1371/journal.pgen.1009646)
Supplement: Attachment — Submitted filename: Point by point letter.docx [file pgen.1009646.s013.docx]

**Point by point letter**

**Editor:**

In particular, we expect that a revised manuscript should:

1. address the role of the catalytic activity of TET1 in mediating chromocenter clustering through the interaction between TET1 and PRC1 complex;

**Response:** We added the chromocenter clustering analysis of the ESCs with forced recruitment of catalytic dead mutant form of TET1 (TET-CD-Mut) to the major satellite repeat (**Fig. 2C and D; line 174-182**). The chromocenter was clustered and its number was significantly reduced even with the recruitment of the catalytically dead mutant form of TET1. We added the discussion in the revised manuscript **(line 284-287).**

2. elaborate on the involvement of PRC1/RING1B on DAPI dense regions in forced TET1 targeting experiments;

**Response:** We analyzed RING1B localization by immunocytochemistry of dCas9-TET1 targeted ESCs (**Fig. S7D and E; line 220-221**). We found that RING1B was significantly enriched in the pericentromeric region of both TET1-CD and TET1-CD-Mut targeting ESCs.

3. clearly describe quantification and statistical methods using in modified histone immunostaining experiments (i.e. H2AK119Ub and H3K27me3) and provide verification of enrichment by another method, such as ChIP. if possible.

**Response:** We analyzed H2AK119ub and H3K27me3 enrichment at major satellite regions by ChIP-qPCR analysis and confirmed the results to immunocytochemistry (**Fig. 3C** and **Fig. S5C**; **line 198-199** and **214-216**).

**Reviewer #1**: Hagihara et al (PGENETICS-D-21-00261)

In this study, the authors described molecular mechanisms of chromocenter clustering at pericentromeric heterochromatin (PCH) using Dnmt1 KO mouse ES cell (mESC) as a model for DNA hypomethylated female meiotic germ cell. Dnmt1 KO DNA hypomethylated mESCs show 5hmC enrichment on PCH and chromocenter clustering (decreased number of DAPI dense foci and HP1g accumulation on them) like female meiotic germ cells. Also, clustered chromocenters in Dnmt1 KO mESCs are enriched for H3K27me3, H2AK119ub, RING1B and TET1. The authors demonstrated that the chromocenter clustering and associating epigenetic changes (5hmC, H3K27me3 and H2AK119ub) are TET1 dependent. Furthermore, the TET1-mediated chromocenter clustering is PRC1 dependent. Finally, the authors showed that the chromocenter clustering is sensitive to 1,6-hexanediol (HD), suggesting that LLPS is involved in this formation in the DNA hypomethylated condition.

Their findings are interesting and provided data are mostly solid. However, if this work focuses on the molecular mechanism of chromocenter clustering in the hypomethylated cells, especially the role of TET1 and PRCs, some of issues remain to be clarified yet. Furthermore, some of different biochemical data should be included for validating their proposal/interpretation. Please tackle following reviewer’s comments in order to improve this manuscript as more complete work.

Major comments

1. Fig. 1A and 3C

a) “The condensed chromocenter of the Dnmt1-KO ESCs retained a strong enrichment of HP1γ, which is similar to meiotic germ cells (Fig. 1A and S1A).” line 125-126.

This finding is interesting. Is this H3K9me3 dependent? Please clarify this. Although this is not a main issue of chromocenter clustering mechanisms, the HP1g enrichment seems to be epigenetically regulated. At least, Fig. 3C data indicates that this phenomenon is chromocenter clustering (compaction?) independent.

**Response:** We agree to the comment that HP1γ deposition at the pericentromeric region in *Dnmt1-*KO ESCs and PGCs likely depends on Suv39h1/h2. Since HP1 family protein localization at pericentromeric region highly relies on Suv39h1/h2 (Saksouk et al., 2014). However, obvious changes in H3K9me3 level between *Dnmt1*-KO and wild-type ESCs was not detected (**Fig. 1A).** This result shows that the hypomethylation-specific HP1γ enrichment at pericentric region is not caused by increased H3K9me3 and is presumably regulated by some unknown factors. We added related information and the discussion in the revised manuscript **(line 316-326).**

b) One more, "HP1γ enrichment at PCH did not change in the Dnmt1/Ring1A/1B-TKO ESCs, suggesting that HP1γ was dispensable for chromocenter clustering under DNA hypomethylated condition. line 213-215.

“HP1g enrichment or accumulation was dispensable” is OK, but “HP1g was dispensable” is say too much. This sentence should be revised.

**Response:** We revised the corresponding part to “HP1γ was insufficient for chromocenter clustering under DNA hypomethylated condition.” **(line 236-238)**.

c) Tet1-KO should be demonstrated by TET1 Ab Western blot analysis.

**Response:** We added the Western blotting results of TET1 in the KO ESCs (**Fig. S2F** and **line 143-146**).

2. Fig. 2 and S2E, F

a) To further validate the dCas9-GCN4/scFvGFP forced recruitment system, the authors should show specific GFP signals of scFv/GFP/TET1-CD on PCH only at the condition of major satellite repeat sgRNA+dCas9-GCN4 but not at scFv-only or dCas9 control as shown in Fig. 2B and C.

**Response:** We added the GFP immunostaining results of scFv-only, dCas9 control ESCs, scFv/GFP/TET1-CD, and scFv/GFP/TET1-CD-Mut and their quantification results (**Fig. 2C** and **S3A**). These results clearly demonstrated the specific recruited of TET1 targeting cassettes to PCH in an sgRNA-dependent manner. To include the FR-TET1-CD-Mut results, we modified the methods (**line 381-386**), and added these results to the revised manuscript (**line 174-175**).

b) “To investigate whether the chromocenter clustering is mediated by the catalytic activity dependent or -independent function of TET1,” line 152-153.

If the authors challenge this problem, the reviewer suggests performing the experiment (Fig. 2) using forced recruitment of TET1-mutantCD (catalytic dead).

**Response:** We added the results of forced recruitment of scFv/GFP/TET1-CD-Mut cassettes to the major satellite regions in the revised manuscript. Representative images, chromocenter counting, GFP quantification, 5hmC staining, and 5hmC enrichment analysis are shown in **Fig. 2 and Fig. S3**. We added these results to the revised manuscript (**line 174-182**).

c) The results of Dnmt1/3a/3b-TKO and Dnmt1/3a/3b/Tet1-QKO ESCs (Fig. S2E and F) are very clear, but at least the statement “the catalytic activity of TET1 are dispensable for chromocenter clustering.” line 160-161 should be revised because these results do not specifically address the catalytic activity of TET1.

**Response:** We revised the manuscript as “Tet1-mediated oxidative products of 5mC, such as 5hmC, 5fC, and 5caC, are dispensable for chromocenter clustering” and also revised corresponding part (**line 155-157** and **162-164**). We also confirmed that Tet1 catalytic activity was not essential for the chromocenter clustering by the forced recruitment experiments (**Fig. 2)**.

d) One more, if essential region of PRC1 (RING1B) interaction in TET1 CD is known, the experiment of forced recruitment of the RING1B interaction defective mutant TET1-CD is also recommended in order to validate whether PRC1 interaction is essential for the forced TET1-CD recruitment-mediated chromocenter clustering. Furthermore, examination of the enrichment of PRC1 (RING1B) on DAPI dense regions by forced recruitment of TET1 CD (WT) in WT ESCs is another suggesting experiment.

**Response:** It is notable that this is the first report showing the interaction between TET1 and PRC1 and that binding sites of TET1 and PRC1 factors had not been identified yet. Thus, the first experiment of this reviewer’s comment is technically too challenging to complete within the reasonable time frame. Instead, following the later suggestions, we added the immunostaining results of RING1B in the scFv/GFP/TET1-CD ESCs to **Fig. S7D and E** and results to the revised manuscript (**line 220-221**).

3. Fig. 3A

H2AK119ub enrichment on DAPI dense regions in Dnmt1 KO ESCs should be validated by H2AK119ub ChIP-qPCR (major satellite repeat). And, Tet1 KO antagonizes this phenotype?

**Response:** We added the ChIP-qPCR results of H2AK119ub to **Fig. 3C** of the revised manuscript (**line 214-216**).

4. Fig. 4B

If Dnmt1 KO specific dynamics of chromocenter rearrangement by 1,6-HD can be observed, it is very nice. If possible, try time-lapse fluorescence microscopic analysis of Hoechst33342 dense foci after the treatment of 1,6-HD (during 1 min). Hoechst33342 can be used for live cell imaging.

**Response:** We agree with this reviewer’s suggestion. However, real-time analysis of 1,6-HD treatment requires the microfluidic system conjugated with confocal microscopy. Unfortunately, our facility does not have the system. We apologize that this experiment is technically impossible in our hands at the moment and appreciate if the reviewer understands the situation.

5. Figure 5 Functional relationship among TEF1, PRC1 and PRC2

Should address following issues.

a) TET1 is essential for PRC1 accumulation on PCH in Dnmt1 KO ESCs, but how about TET1 enrichment on PCH in Dnmt1/Ring1A/1B-TKO ESCs?

**Response:** We added the TET1 immunostaining results of *Dnmt1/Ring1A/1B*-TKO ESCs to **Fig. S9B and C**. The enrichment of TET1 at the chromocenter was slightly but significantly reduced in the *Dnmt1/Ring1a/b*-TKO ESCs, suggesting that TET1 localization was partly regulated by PRC1. We added these results to the revised manuscript (**line 238-240**).

b) How about PRC1, H2AK119ub, TET1 and HP1g enrichment on PCH in Dnmt1/Ezh2-DKO ESCs?

These results further clarify functional relationship among them for chromocenter clustering.

**Response:** We analyzed the enrichment of RING1B (**Fig. S8A and B**), H2AK119ub (**Fig. S8C and D**), TET1 (**Fig. S6C and D**), and HP1γ (**Fig. S6E and G**) in *Dnmt1/Ezh2*-DKO ESCs through immunostaining. We added these results to the revised manuscript (**line 206-207**, **209-210,** and **221-225**).

6. Fig S4A

The Tet1 and Dnmt1-KO dependent H3K27me3 PCH enrichment should be validated by H3K27me3 ChIP-qPCR. Also, examine EZH2 enrichment on them, at least by immunostaining.

**Response:** We added the ChIP-qPCR results of H3K27me3 in *Dnmt1*-KO and *Dnmt1/Tet1*-DKO ESCs to **Fig. S5C**. Also, we added the EZH2 immunostaining results to **Fig.S5D and E** and revised manuscript (**line 198-201**).

7. Fig. S5A

The Tet1 and Dnmt1-KO dependent RING1B enrichment on DAPI dense regions should be quantitatively measured and validated statistically.

**Response:** We added the results of RING1B quantification analysis to **Fig.S7B** and revised manuscript (**line 216-217**).

8. Fig. S6

Control data (no HD treated WT cell) should be included.

**Response:** We added the no-treatment WT ESCs to **Fig.S10A and B** of revised manuscript.

Minor comments,

9. The authors should discuss (at least one sentence) whether the NANOG pathway interacts with the TET1-PRC1 pathway for chromocenter clustering in mESCs.

**Response:** We added Western blotting and immunofluorescent analysis of NANOG, and found it was not affected in neither *Dnmt1*-KO nor *Dnmt1/Tet1*-DKO ESCs (**Fig. S4**). We added these results to the revised manuscript (**line 185-189**).

10. hKO1-HP1γ

Need more explanation. What is hKO1.

**Response:** We thank the reviewer for noting the missing points in our manuscript. We revised the manuscript (**line 248-249)**.

11. “The CXXC domain, which can bind to unmethylated DNA, is present in TET1 but not in TET2, and this difference may account for the difference in PCH targeting.” line 257-259.

The authors should cite original work if it is already known that the CXXC domain of TET1 binds to unmethylated DNA.

**Response:** We added the reference (**line 284**).

12. P14 line 268. Fig. S3A, S3B→Fig. S4A, S4B?

**Response:** We apologize for this mistake. The manuscript is corrected in the revised manuscript (**line 294**).

Reviewer #2: How histone H3K27me3 compensates the loss of H3K9me2/3 or DNA methylation at pericentromeric heterochromatin (PCH) has puzzled this field for a long time. In this manuscript, Hagihara et al. showed mostly by immunostaining that TET1 plays a central role in the recruitment of polycomb repressive complexes to reorganizing chromocenters in Dnmt1 knockout mouse embryonic stem cells. First, they revealed that TET1-depletion reverted the clustered chromocenters in Dnmt1 KO mouse ES cells to scattered smaller ones. Then, they validated the effect of TET1 by ectopically tethering the catalytic domain of TET1 to PCH and found that forced recruitment of TET1-CD was sufficient to induce chromocenter clustering. PRC1 and PRC2 have been shown to localize to PCH in response to DNA hypomethylation. The authors further showed that PRC1 was critical for chromocenter clustering and the deposition of H2AK119ub was dependent on the presence of TET1. Finally, with time-lapse imaging and chemical perturbation, the authors found that the chromocenter clustering in Dnmt1 KO mouse ES cells was driven by liquid-liquid phase separation.

This manuscript provided very clear results with high quality. The demonstration for the essential role of TET1 in mediating chromocenter clustering in Dnmt1 KO cells is convincing. The authors proposed that the underlying mechanism is through the interaction between TET1 and PRC1 complex and indispensable on the enzymatic activity of TET1. However, there is no direct evidence against the involvement of TET1's catalytic activity in the manuscript. Tethering a catalytic-dead form of TET1-CD to PCH to examine its effect on chromocenter clustering will help to clarify this.

**Response:** We analyzed the effects of the artificial tethering of scFv/GFP/TET1-CD-Mut to the major satellite repeat regions, and confirmed that chromocenter clustering was mediated in a catalytic activity-independent manner (**Fig. 2 and S3)**. We added these results to the revised manuscript (**line 178-182**).

Another major concern is that the authors tried to associate the observations on the function of TET1 in Dnmt1 KO cells with that in meiotic female germ cells regardless of inconsistent features. In their previous publication (Nature, 2012), they revealed that Tet1 deficiency resulted in no significant change in HP1γ staining in the oocyte. The current study, on the contrary, showed in the first main figure that Tet1 deletion diminished HP1γ signals at DAPI-dense chromocenters in Dnmt1 KO cells, suggesting the PCH organization mechanism in DNA hypomethylated ES cells is quite different from that in female germ cells. The authors need to revise their manuscript and reconsider their statements related to the phenomenon in female meiosis. In addition, the authors showed that PCH in female germ cells was devoid of H3K9me2 in Fig. S1B. Is it also true in Dnmt1 KO ES cells?

**Response:** We thank this reviewer for the insightful comments. We found HP1γ enrichment at hypomethylated ESCs depends on *Tet1*, whereas it was independent of *Tet1* in female gonocytes. In addition, we analyzed H3K9me2 enrichment by immunostaining and found that H3K9me2 was not affected in *Dnmt1*-KO and *Dnmt1/Tet1*-DKO ESCs (**Fig. S1C and D; line 126-128)**. Thus, we believe that these findings are potentially interesting to understand the epigenetic hierarchy of pericentromeric heterochromatin. We added the discussion to the revised manuscript (**line 316-326**).

Minor concern:

In Lines 214 and 215, the authors concluded that "HP1γ was dispensable for chromocenter clustering under DNA hypomethylated condition." From Fig. 3C, HP1γ still localized to DAPI-dense chromocenters in Dnmt1/Ring1a/Ring1b triple knockout cells and the chromocenter clustering was ameliorated. This result only indicated that HP1γ was insufficient for chromocenter clustering rather than dispensable.

**Response:** We completely agree with this reviewer, and the corresponding part was changed following this suggestion in the revised manuscript (**line 236-238**).
